# Effect of the traditional Chinese medicine Pinggan-Qianyang decoction on SIRT1–PTEN signaling in vascular aging in spontaneously hypertensive rats

**DOI:** 10.1038/s41440-021-00682-6

**Published:** 2021-06-29

**Authors:** Zhang Cui, Yao Jiamei, Yang Yushu, Fang Xia, Yang Haiyan, Dan Zhang, Chen Qiong, Zhong Guangwei

**Affiliations:** 1 International Medical Center, Xiangya Hospital, Central South University, Changsha, Hunan 410008 P.R. China; 2 National Clinical Research Center for Geriatric Disorders, Xiangya Hospital, Central South University, Changsha, Hunan 410008 P.R. China

**Keywords:** Spontaneously Hypertensive Rat, Pinggan-Qianyang decoction, Silent information regulator 1 (SIRT1), Phosphatase and tensin homology deleted on chromosome ten (PTEN), Vascular aging

## Abstract

Age-related functional decline is a physiological phenomenon that occurs in all organ systems. However, the acceleration and early occurrence of this process are observed in cardiovascular pathologies, including hypertension. This study aimed to investigate SIRT1–PTEN signaling in aortic tissue from spontaneously hypertensive rats (SHRs) and changes in SIRT1 and PTEN expression following treatment with Pinggan-Qianyang decoction (PGQYD) and explore the mechanism involved in the treatment of hypertensive vascular aging with traditional Chinese medicine. In this study, we used two rat models: spontaneously hypertensive rats (SHRs) at 14 and 64 weeks of age and WKY rats at 64 weeks of age. The degree of irritability and rotation tolerance time were evaluated to determine the effects of PGQYD on animal behavior. The morphology of the thoracic aorta was examined by hematoxylin-eosin (HE) and Masson staining and electron microscopy. Nicotinamide adenine dinucleotide phosphate (NADPH) oxidase activity and superoxide dismutase (SOD) and anti-superoxide anion content were detected. Senescence-associated β-galactosidase (SA-β-Gal) staining was used to observe the thoracic aorta during vascular aging. RT-qPCR, immunofluorescence, and Western blot analysis were performed to detect changes in the mRNA and protein expression of p53, p21, SIRT1, and PTEN in rat aortic tissues. Behavioral tests and histological and morphological analyses showed the remarkable amelioration of vascular aging after PGQYD treatment compared with that in the older SHRs. Moreover, PGQYD significantly decreased vascular aging in SHRs, as indicated by reduced SA-β-Gal staining, NADPH oxidase activity, and p53 and p21 expression, and increased anti-superoxide anion and SOD content. Furthermore, PGQYD increased SIRT1 and PTEN expression, but the downregulated expression of SIRT1 induced by a SIRT1 inhibitor abolished the PGQYD-induced antiaging effects on gene expression and antioxidant activity and enhanced PTEN expression. PGQYD could ameliorate vascular aging effects in SHRs, which may have been mediated via the regulation of SIRT1–PTEN signaling in aortic tissue.

## Introduction

The aging of vascular smooth muscle cells (VSMCs) is the main pathological mechanism underlying cardiovascular and cerebrovascular diseases, such as vascular aging, hypertension, and atherosclerosis [1]. Senescent VSMCs can change the local tissue microenvironment, promoting the occurrence of inflammation and vascular sclerosis [2] and aggravating the development of atherosclerosis and hypertension, by secreting a variety of cytokines and growth factors. In-depth studies on the occurrence of vascular aging and mechanisms for its prevention are lacking at present.

Silent information regulator 1 (SIRT1), an NAD+-dependent histone deacetylase, plays an important role in gene silencing, inhibiting DNA recombination, and prolonging cell lifespan when energy is limited [3]. Recent studies have shown that SIRT1 is critically involved in delaying VSMC aging and improving vascular function through its anti-inflammatory, antioxidative stress, antiapoptotic, and other protective mechanisms [4]. In addition to its activity against histones as substrates, SIRT1 can interact with a variety of transcription factors including p53, FOXO1, STAT3, PGC-1α, and PTEN to regulate their transcriptional regulation activity [5]. Of these transcription factors, PTEN, one of the earliest identified tumor suppressor genes with phosphatase activity, is related to the regulation of various physiological activities and processes, such as the cell cycle, apoptosis, adhesion, migration, and vascular growth [6]. PTEN can both inhibit the proliferation and migration of VSMCs and promote VSMC apoptosis [7].

We previously reported the use of Pinggan-Qianyang decoction (PGQYD), which can improve vascular remodeling and blood pressure circadian rhythm, to treat essential hypertension [8]. PGQYD is also effective in preventing and reversing hypertension-related vascular remodeling [9]. The purpose of this study was to investigate whether PGQYD can delay the aging of hypertensive blood vessels and to conduct a preliminary exploration of its possible mechanism of action to provide a theoretical basis for the treatment of essential hypertension using PGQYD.

## Materials and methods

### Main experimental reagents and instruments

TRIzol was purchased from Invitrogen (United States). DEPC was obtained from Sigma (United States). Selisistat (EX 527), a specific SIRT1 inhibitor, was purchased from Selleck Chemicals (United States). ReverTra Ace-α-^TM^ reverse transcription kits were obtained from Shanghai TOYOBO Biological Co., Ltd. GoTaq Green Master Mix (2×) was purchased from Beijing Dingguo Changsheng Biotechnology Company. IDNA Ladder, a set of molecular markers, was obtained from Beijing Solarbio Company. Rabbit anti-rat p53, anti-p21, anti-SIRT1, anti-PTEN and anti-rat GAPDH monoclonal antibodies were purchased from Cell Signaling Technology, Inc. (United States). An anti-superoxide anion detection kit (A052-1-1), nicotinamide adenine dinucleotide phosphate (NADPH) kit (A127-1-1), and SOD kit (A001-3-2) were purchased from the Nanjing Jiancheng Biology Engineering Institute.

Two physiological recorders (type LMS-2B) were provided by Chengdu Instrument Factory. Blood pressure monitors for conscious animals (type HX-III, patent number: 892118741) were provided by the Department of Cardiac Physiology Laboratory at Xiangya Medical College, Central South University. A Tanon GIS-2020 electrophoresis gel image analyzer was provided by Shanghai Tianneng Technology Co., Ltd. A type 5332 PCR amplifier was provided by Eppendorf (Germany). All primers were synthesized by Shanghai Sangon Bioengineering Technology Company.

### Animal grouping and treatment

Sixty-four-week-old male spontaneously hypertensive rats (SHRs; *n* = 30, clean-grade, weighing 240 ± 10 g) were provided by Shanghai Slack Laboratory Animal Co., Ltd. (license number: SCXK (Shanghai) 2003-0003). Sixty-four-week-old male WKY rats (Normal group, *n* = 10) and 14-week-old male SHRs (Youth group, *n* = 10, clean-grade, weighing 210 ± 10 g) were provided by the Animal Experiment Center of Central South University (license number: 20-010). All rats were acclimatized for 2 weeks in the laboratory before the experiment. All animal experiments were approved by the Animal Care and Use Committee of Central South University.

The experimental groups were treated as follows: ① WKY rats in the Normal group (*n* = 10, aged 64 weeks) were continuously given saline for 49 days; ② SHRs in the Youth group (*n* = 10, aged 14 weeks) were continuously given saline for 49 days; ③ SHRs in the Old group (*n* = 10, aged 64 weeks) were continuously given saline for 49 days; ④ SHRs in the TCM group (*n* = 10, aged 64 weeks) continuously received PGQYD (by intragastric administration) for 49 days; ⑤ and SHRs in the SIRT1 inhibitor-treated (EX 527 + TCM) group [*n* = 10, aged 64 weeks] were continuously given EX 527 (10 mg/kg per day, intraperitoneal administration) and PGQYD (intragastric administration) for 49 days. Based on the equivalent dose between humans and animals, which was determined with the formula S rats/200 g = 0.018 * S human/70 kg, the experimental drug was dissolved in water and given at the appropriate dose by gavage at a rate of 1 mL/100 g [10].

### Drug preparation

PGQYD is composed of five medicinal herbs: Tian Ma (*Gastrodiae Rhizoma*), Gou Teng (*Uncaria rhynchophylla*), Shi Jue Ming (*Haliotis discus hannai)*, Mao Li (*Ostrea gigas* Thunberg), and Niu Xi (*Cyathulae Radix*) at a ratio of 10:20:30:30:20 (dry weight). All herbs were obtained from Xiangya Hospital at Central South University (CSU) (Changsha, China). Then, each herb was authenticated by Professor Shao Liu, an herbal medicinal botanist at the Department of Pharmacy in Xiangya Hospital, CSU. The decoction was prepared and subjected to quality control assessment as previously described [11]. Finally, the powder was dissolved in distilled water at a concentration of 0.2 g/ml for intragastric administration.

### Behavioral observation and blood pressure measurements

Irritability was observed and scored as follows [12]: animals that squealed and were startled when held by the neck, 1 point; animals that were agitated and tried to bite when held by the neck, 2 points; animals that squealed, were startled and tried to bite the rats in the same cage or fought frequently and often bit the iron cage when their tails were raised, 3 points. A score of zero was given if none of the above descriptions clearly applied. One more measurement was made before the end of the experiment.

Rotation tolerance time was measured as follows: the rat was placed on a homemade platform rotating at 45 rpm. The rotation time was the time until the rate fell once placed on the platform. If the rat did not fall down after rotating for 2 min, the experiment was stopped, and one more measurement was made before the end of the experiment.

Blood pressure was assessed as follows: the systolic blood pressure of the rats was measured three times before treatment and on the 7th, 21st, 35th, and 49th days of the experiment with the tail artery pressure method as reported by Yang et al. [13]. The average value for the day was recorded.

### Histological observation of the thoracic aorta

Rats were anesthetized with an intraperitoneal injection of 5 ml/kg 20% urethane at the end of each week of all-day drug administration. The thoracic aorta below the aortic arch of each rat was stripped and clipped [14]. A portion was fixed in 8% neutral formaldehyde, embedded in paraffin, sectioned at 5 µm, stained with HE and Masson trichrome, and examined by electron microscopy. Light microscopy was used to image each cross-sectional slice (five per rat). Each vascular ring in the perpendicular position and the vessel media wall were observed. Images were observed under a Leica imaging system (Leica Microsystems GmbH, Wetzlar, Germany). Media thickness (MT) and lumen diameter (LD) was measured, and the ratio of MT/LD calculated [14].

### Detection of vascular aging-related indicators

To determine the oxidative stress index, NADPH oxidase activity in the thoracic aorta was detected with an assay kit (with a colorimetric method). The plasma SOD level was detected with an assay kit (WST-1 method).

To determine the anti-superoxide anion content in thoracic aortas from the rats in each group, 10% of the supernatant of homogenized vascular tissue was sampled to detect the level of reactive oxygen species with a kit according to the instructions (Nanjing Jiancheng Biology Engineering Institute).

### SA-β-Gal staining

SA-β-Gal staining was performed using a senescence-associated β-galactosidase staining kit (CBA-230; Cell Biolabs Inc., San Diego, CA, USA) according to the manufacturer’s protocol. Briefly, sections of the thoracic aorta were fixed in a β-galactosidase fixation solution (2% formaldehyde/0.2% glutaraldehyde in phosphate-buffered sulfate (PBS)) for 10 min and then washed three times with PBS. The sections were stained in SA-β-gal staining solution (pH 6.0) overnight at 37 °C. The number of cells positive for senescence-associated β-galactosidase (SA-β-gal) was observed by light microscopy (Olympus CK40, Japan) in 10 randomly chosen low-power fields (×200), and the data are expressed as the percentage of the counted cells.

### Immunofluorescence analysis

Frozen, acetone-fixed thoracic aorta sections (5 µm) were incubated for 1 h in 10% bovine serum to block nonspecific protein–protein interactions. Then, they were incubated overnight at 4 °C in PBS with primary antibodies (rabbit anti-rat PTEN antibody, 1:200; Cell Signaling Technology, Inc. and rabbit anti-rat SIRT1 antibody, 1:200, Cell Signaling Technology, Inc.). After washing three times for 10 min, the sections were incubated in PBS for 1 h at room temperature with a secondary antibody (goat anti-rabbit Alexa Fluor 594, 1:200). Nuclei were counterstained with 4′-6-diamidino-2-phenylindole (DAPI; Invitrogen, Carlsbad, CA, USA). Immunofluorescence staining was analyzed using a laser-scanning confocal microscope (SLM 510, Carl Zeiss Meditec, Inc., Jena, Germany).

### Real-time quantitative PCR analysis

Total RNA was extracted from thoracic aorta tissue samples from rats in the four groups (Youth, Old, TCM, and EX 527 + TCM) using TRIzol reagent (Thermo Fisher Scientific) according to the manufacturer’s instructions. cDNA was generated using the High-Capacity cDNA Reverse Transcription Kit (Thermo Fisher Scientific). RT-qPCR was performed using the Stratagene Mx3005P RT-qPCR System (Applied Biosystems) and PowerUp^TM^ SYB^R^ Green Master Mix (Thermo Fisher Scientific) according to the protocol. Melt curves were analyzed at the end of each assay to confirm specificity. Fold changes in expression were determined using the 2-CT method, and data were normalized using the levels of the endogenous control, *GAPDH*. The PCR primers used are listed in Supplementary Table S1.

### Western blot analysis

Proteins were extracted from frozen thoracic aorta tissue samples, and protein concentrations were measured using a bicinchoninic acid protein assay. The samples were incubated with primary antibodies (rabbit anti-rat p53, anti-p21, anti-SIRT1, and anti-PTEN monoclonal antibodies (1:200) and rabbit anti-rat GAPDH monoclonal antibody, 1:300). After washing with TBST, the secondary antibody (1:200) was added, and the blots were incubated at room temperature for 3 h before being washed with TBS again. ECL developing solution was added to membranes, which were finally exposed to X-ray film for 5–8 min. Quantity One analysis software (Bio-Rad, Hercules, CA) was used to analyze the results, and the integrated optical density (IOD) value was obtained by dividing the level of the target protein by that of the internal reference band (GAPDH) and used as the final result for statistical analysis. The primary antibodies used are listed in Supplementary Table S2.

### Statistical analysis

Statistical analysis was performed using SPSS 18.0 software. Differences between continuous variables were assessed by variance analysis (ANOVA), and the data are expressed as the mean ± standard error of the mean (${\bar{\mathrm{X}}}$ ± SEM). Rank count data were tested using a rank-sum test. Differences between categorical variables were tested using the chi-square test. The test level was *α* = 0.05 (two-sided), and *P* < 0.05 was used to indicate statistical significance.

## Results

### Effects of PGQYD on behavior and blood pressure in SHRs

**Fig. 1:**
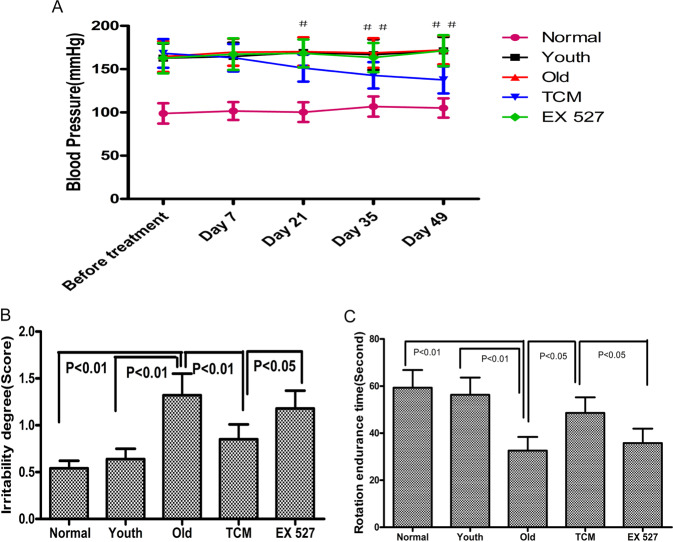
Effect of PGQYD on behavior and blood pressure in SHRs. **A** Comparison of rat systolic blood pressure. **B** Comparison of rat irritability. **C** Comparison of rat rotation tolerance times. Data are presented as the mean ± SEM (*n* = 10 per group). *P < 0.05 and **P < 0.01, Old group versus TCM group. ^#^*P* < 0.05 and ^##^*P* < 0.01, TCM group versus EX 527 group

As shown in Fig. 1B, C, compared with the Normal group and Youth group, the Old group showed a significantly higher degree of irritability and dramatically shorter rotation tolerance time (*P* < 0.01). Furthermore, compared with the Old group, the TCM group showed a significant decrease in the degree of irritability (*P* < 0.05) and markedly prolonged rotation tolerance time (*P* < 0.05), qualities that are essentially consistent with the clinical manifestations of liver-Yang hyperactivity syndrome in traditional Chinese medicine. These results demonstrated that PGQYD could improve the behavior of the SHRs.

As shown in Fig. 1A, before treatment, blood pressure in the four groups of SHRs did not differ significantly (*P* > 0.05). In SHRs and WKY rats, blood pressure differed greatly. Among the SHRs, blood pressure in the Youth group and Old group did not differ significantly (*P* > 0.05). Compared with that in the Old group, systolic blood pressure in the TCM group was reduced after the third week (*P* < 0.05) and stable over the rest of the treatment period (in the third, fifth, and seventh weeks).

Pretreatment with the specific SIRT1 inhibitor EX 527 abrogated the PGQYD-mediated antihypertensive effect and amelioration of behavioral changes (the degree of irritability and rotation tolerance time) in the SHRs. Taken together, these results indicate that PGQYD reduced systolic blood pressure and ameliorated the behavior of SHRs via SIRT1 signaling.

### Effect of PGQYD on vascular aging in SHRs, as assessed based on morphology

**Fig. 2:**
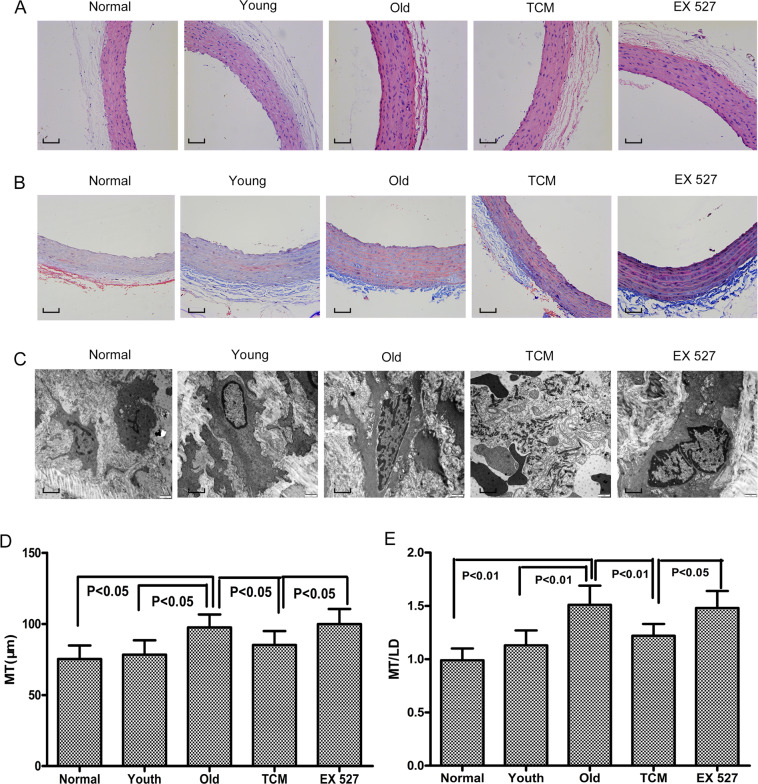
Effect of PGQYD on SHR thoracic aorta morphology. **A** HE staining of the rat thoracic aorta. **B** Masson staining of the rat thoracic aorta. **C** Electron microscopy observation of rat thoracic aortic vascular tissue. **D** Comparison of rat thoracic aorta media thickness (MT). **E** Comparison of rat thoracic aorta media thickness to luminal diameter ratio (MT/LD). Data are presented as the mean ± SEM (*n* = 10 per group)

Masson and HE staining showed that the aortic tunica media of the Old group was thicker than that of the Normal group and Youth group, while that of the PGQYD-treated rats was thinner than that of rats in the Old group (Fig. 2A, B). As shown in Fig. 2D, E, both the MT and MT/LD were higher in the Old group than in the Normal group and Youth group (*P* < 0.01); however, both the MT and MT/LD were significantly lower in the TCM group than in the Old group (*P* < 0.01 or *P* < 0.05). Compared with those in the TCM group, the aortic tunica media was thicker and the MT and MT/LD were clearly increased in rats administered EX 527 intrathecally.

The results of aortic electron microscopy analysis of the rats in each group are presented in Fig. 2C. In the Normal group and Youth group, the organelles in the cytoplasm of VSMCs were not well developed, few mitochondria were seen, and the endoplasmic reticulum was visible. However, in the Old and EX 527 groups, organelles in the cytoplasm of VSMCs were more developed, more mitochondria were visible, and the endoplasmic reticulum was more visible. In the TCM group, organelles in the VSMCs were more developed, with visible mitochondria and endoplasmic reticulum. These data suggest that PGQYD can improve morphological changes in the aortic vascular tissue and reverse vascular aging in SHRs via SIRT1 signaling.

### Effect of PGQYD on biomedical indicators related to vascular aging in SHRs

**Fig. 3:**
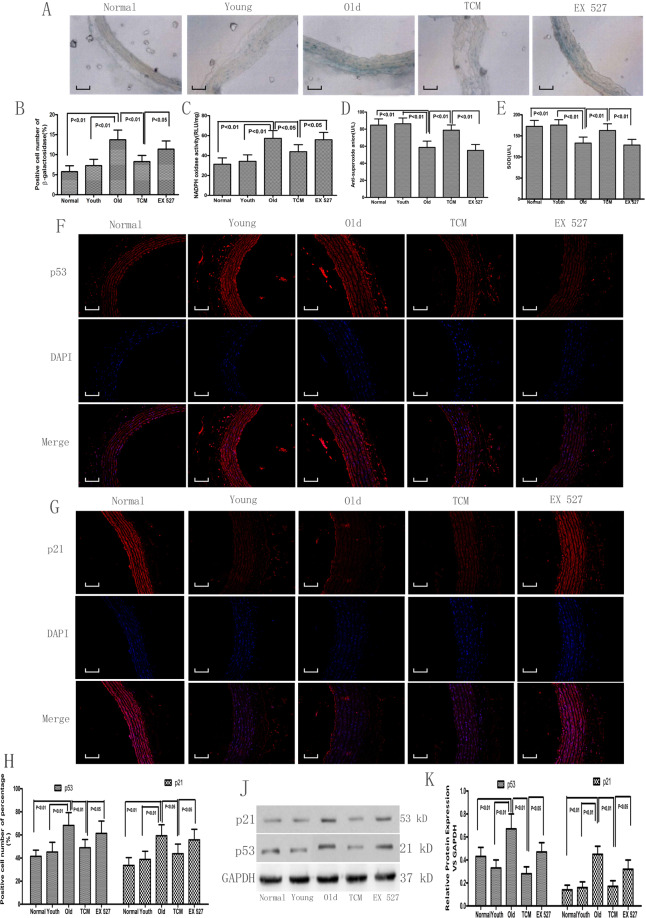
Effect of PGQYD on vascular aging in SHRs. **A**, **B** Thoracic aorta vascular aging was observed by SA-β-Gal staining. **C**–**E** The plasma SOD content, NADPH oxidase activity, and anti-superoxide anion content in the aortic tissue of SHRs were determined. **F** Representative images showing immunofluorescence staining of p53 and quantification of the p53-positive area. **G** Representative images showing immunofluorescence staining of p21 and quantification of the p21-positive area. **H** Semiquantitative analysis of p53 and p21 protein expression. **J**, **K**. The quantitative expression of p53 and p21 was detected by Western blot analysis. Data are presented as the mean ± SEM (*n* = 10 per group). Panels show DAPI nuclear staining (blue, middle panels), staining with the corresponding antibody (SIRT1/PTEN) (red, left panels), and merged images (right panels). Original magnification: ×200. DAPI, 4′,6-diamidino-2-phenylindole; EX 527, a specific SIRT1 inhibitor; TCM traditional Chinese medicine

Vascular aging is a major independent risk factor for cardiovascular diseases, including coronary heart disease, stroke, and essential hypertension. Therefore, we examined senescence-associated β-galactosidase activity by using SA-β-gal as a marker (Fig. 3A, B) and senescence-related protein expression by immunofluorescence analysis (Fig. 3F–H) and western blot analysis (Fig. 3J, K). After TCM treatment, the expression of p53 and p21 and SA-β-Gal staining rate were significantly lower than those of the Old group. Moreover, EX 527 reversed the PGQYD-induced downregulation of p53 and p21 expression and decrease in SA-β-Gal staining rate in the SHRs. Thus, these results suggest that PGQYD can reverse vascular aging through SIRT1 signaling.

Oxidative stress is a key inducer of cell senescence and aging-associated diseases. Thus, we next aimed to further determine the effect of PGQYD on senescence-associated oxidative stress in hypertension. Representative images (Fig. 3C–E) show that PGQYD induced a remarkable increase in the anti-superoxide anion and SOD content and decreased NADPH oxidase activity. Moreover, the effects of PGQYD were abolished by pretreatment with the SIRT1 inhibitor EX 527. Collectively, these results indicate that PGQYD inhibits oxidative stress via the activation of SIRT1 signaling.

### Effect of PGQYD on SIRT1–PTEN signaling in SHRs

**Fig. 4:**
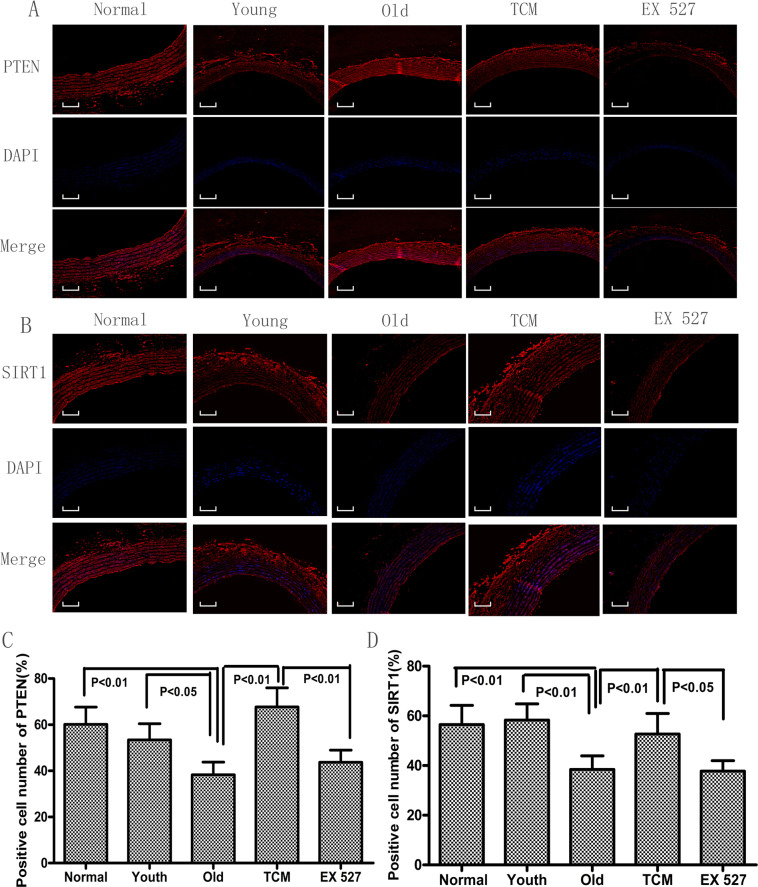
Immunofluorescence staining of SIRT1 and PTEN in vascular tissue. **A** Representative image showing immunofluorescence staining of SIRT1 and quantification of the SIRT1-positive area (*n* = 10). **B** Representative images showing immunofluorescence staining of PTEN and quantification of the PTEN-positive area (*n* = 10). Data are presented as the mean ± standard error of the mean. Panels show DAPI nuclear staining (blue, middle panels), staining with the corresponding antibody (SIRT1/PTEN) (red, left panels), and merged images (right panels). Original magnification: ×200. DAPI, 4′,6-diamidino-2-phenylindole; EX 527, a specific SIRT1 inhibitor; TCM, traditional Chinese medicine. **C** Quantitative analysis of expression of the aging-related protein PTEN, as detected by fluorescence immunohistochemical analysis. **D** Quantitative analysis of expression of the aging-related protein SIRT1, as detected by fluorescence immunohistochemical analysis.

As shown in Fig. 4, the PTEN and SIRT1 proteins were expressed at lower levels in the cytoplasm around the nucleus in the Old group compared to the Normal and Youth groups; however, in the TCM group, SIRT1 and PTEN protein levels were significantly increased compared with those in the Old group. We also observed that PGQYD-mediated upregulation of PTEN and SIRT1 was diminished by the intrathecal administration of EX 527. The results of immunofluorescence assays were consistent with those of the western blot analyses.

**Fig. 5:**
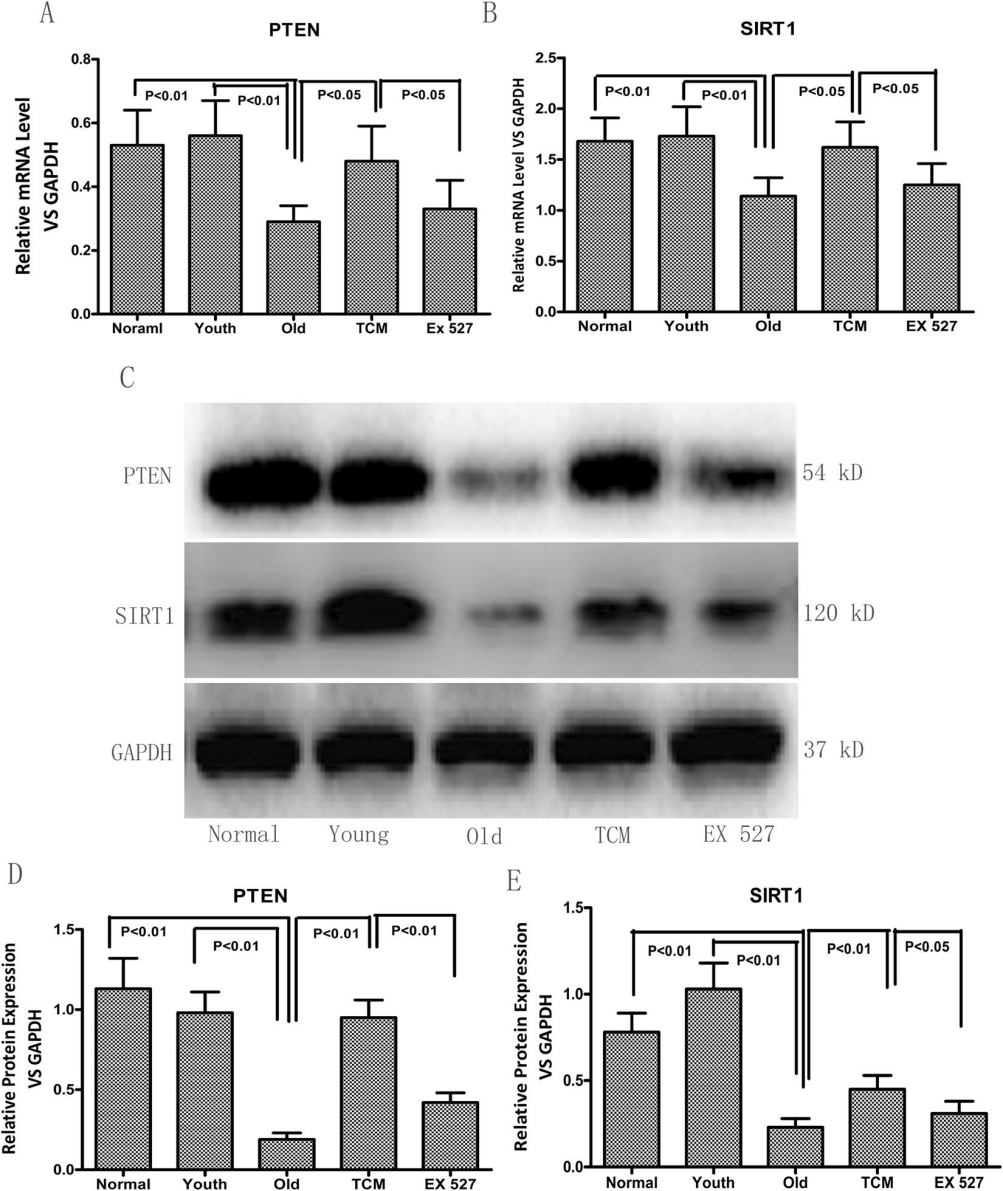
Effect of PGQYD on the quantitative expression of aging-related genes and proteins in SHRs. **A** *PTEN* mRNA expression detected by RT-qPCR. **B** Expression of *SIRT1* mRNA detected by RT-qPCR. **C** Expression of aging-related proteins detected by western blot. **D** Quantitative analysis of expression of the aging-related protein PTEN, as detected by western blot analysis. **E** Quantitative analysis of expression of the aging-related protein SIRT1, as detected by western blot analysis. Data are presented as the mean ± SEM (*n* = 10 per group)

As shown in Fig. 5, SIRT1 gene and protein expression in the Old group was significantly downregulated relative to that in the Normal group and Youth group, while PTEN gene and protein expression was remarkably downregulated. Compared with those in the Old group, PTEN and SIRT1 gene and protein levels were significantly increased in the TCM group; however, the SIRT1 and PTEN gene and protein expression levels differed significantly between the EX 527 and TCM groups. Taken together, our findings suggest that PGQYD can regulate the expression of SIRT1 and PTEN, which are related to vascular aging, in SHRs through the regulation of SIRT1 signaling.

## Discussion

In traditional Chinese medicine, hypertension belongs to a category of diseases and syndromes that include “dizziness” and “headache”. After summarizing and analyzing clinical research literature on hypertension, Chinese medical scientists believed that hypertension is based mainly in the liver and that its symptoms are mainly caused by liver—Yang hyperactivity syndrome [15]. Our previous studies demonstrated that PGQYD can both reduce blood pressure and improve and reverse vascular remodeling in hypertension, thereby delaying vascular senescence [8, 9]. The results of the present study showed a significant and sustained decrease in blood pressure at each time point after treatment with PGQYD. In addition, morphological observation revealed that PGQYD could clearly inhibit structural changes related to vascular aging in hypertension. Our data also demonstrate that PGQYD can increase anti-superoxide anion and plasma SOD levels and reduce NADPH oxidase activity in vascular tissue, suggesting that PGQYD can reduce blood pressure and delay vascular aging associated with hypertension and that the mechanism may involve the adjustment of parameters related to oxidative stress.

In normal human cells, inactivation of the p53 or p21 gene can prolong the replicative lifespan of cells, suggesting that this pathway is closely related to cell senescence [16]. p53 mediates the response of cells to DNA damage. In addition, p53 can mediate not only replicative senescence caused by telomere shortening but also premature aging caused by stress [17]. Its downstream target molecule is p21, an inhibitor of cyclin-dependent protein kinases, which can inhibit the cell cycle and cause irreversible growth arrest [18]. p21, a broad-spectrum cell-cycle inhibitor, can cause G1-phase arrest and G2-phase arrest by specifically inhibiting the protein kinase activities of cyclin D1-CDK4/CDK6, cyclin E-CDK2, and cyclin A-CDK [19]. Gastrodin can inhibit the proliferation of VSMCs and delay aging [20]. The total alkaloids of *U. rhynchophylla* could inhibit the proliferation of endothelial cells induced by Ang II in a dose-dependent manner and inhibit the senescence of endothelial cells induced by β-galactose [21]. Ginseng (*Panax notoginseng*) chuanxiong extract could improve aging-associated changes in aortic morphology in aging mice; reduce the generation of reactive oxygen species and AGEs in vascular tissue; inhibit the activity of MMP-2; regulate the MMP-2/TIMP-2 balance, ultimately reducing vascular stiffness in aging mice; reduce vascular remodeling, and delay the occurrence of vascular aging in mice [22]. The data in our study demonstrate that PGQYD can decrease the expression of p53 and p21 in senescent vascular tissues. This suggests that PGQYD can reduce the occurrence of vascular tissue aging, which may be closely related to its inhibitory effect on the expression of p53 and p21.

A recent study reported the sirtuin protein SIRT1 to be a new selective substrate of nuclear autophagy in senescence and aging [23]. Upon the senescence of primary human cells, SIRT1 degradation is mediated by the direct nuclear SIRT1–LC3 interaction, followed by nucleus-to-cytoplasm shuttling of SIRT1 and autophagosome–lysosome degradation. In vivo, SIRT1 was found to be downregulated by lysosomes in hematopoietic and immune organs upon natural aging in mice and in aged human T cells. These results revealed another substrate of nuclear autophagy and suggest a new strategy to promote SIRT1-mediated health benefits by suppressing autophagic SIRT1 degradation. Ginsenosides have been reported to act as SIRT1 activators. Importantly, many ginsenosides can be used to prevent and treat oxidative stress, inflammation, aging, tumorigenesis, depression, and other conditions by targeting the SIRT1 signaling pathway [24]. Moreover, other investigations have suggested that increased SIRT1 activity can prevent aging [25, 26]. Therefore, based on previous reports, the appropriate upregulation or activation of SIRT1 has emerged as a promising method for the treatment of vascular senescence and age-related cardiovascular disease [27]. Our data demonstrate that PGQYD can increase the expression of SIRT1 in senescent vascular tissues. This result suggests that SIRT1 plays an important role in the ability of PGQYD to delay vascular aging in hypertension.

Recent reports showed that *PTEN* transgenic mice presented increased energy expenditure, decreased adiposity, improved insulin sensitivity upon high-fat feeding or with aging, and an extended lifespan [28]. These findings have led to new mechanistic insights into the role of PTEN in metabolism. Interestingly, PTEN promotes oxidative phosphorylation and decreases glycolysis, thus preventing characteristic metabolic reprogramming in cancer cells, which might be relevant to PTEN-mediated protection against aging [29]. PTEN was also found to upregulate UCP1 expression in brown adipocytes, enhancing their nutrient-burning capacity and decreasing adiposity and associated pathologies [30]. These newly discovered effects of PTEN on metabolism open new avenues for explorations relevant to cancer, obesity, diabetes, and aging [31]. Our data demonstrated that PGQYD could increase the expression of PTEN in senescent vascular tissues. This finding suggests that PTEN plays an important role in the ability of PGQYD to delay vascular aging in hypertension.

SIRT1 can regulate the expression of PTEN [32]. To further explore this finding, Wang et al. modeled the structures of SIRT1 and PTEN with data from the RCSB PDB database and then docked SIRT1 onto PTEN with MOE software. The use of MOE software docked PTEN to the C‐terminal domain of SIRT1. Hydrogen bonds at the interface between SIRT1 and PTEN were found, which indicated that SIRT1 can interact with PTEN and directly regulate its expression. Resveratrol inhibited oxidative stress and reversed methamphetamine-induced increases in permeability and apoptosis of the alveolar epithelium by activating the SIRT1/PTEN/p-Akt pathway [33]. Further in vitro studies demonstrated that the inhibition of Sirtuin 1 (SIRT1) activity with a SIRT1 inhibitor and RNA interference decreased PTEN protein expression, while resveratrol attenuated the decrease in PTEN expression induced by the SIRT1 inhibitor [34]. The data in our study demonstrated that EX 527, an inhibitor of SIRT1, could inhibit PTEN expression and reverse the PGQYD-mediated upregulation of PTEN. Tianma Gouteng decoction was found to regulate the autophagy-related Ca^2+^/AMPK/mTOR pathway in the vascular endothelial cells of SHRs, which is most likely an important mechanism responsible for its antihypertensive effect [35]. Gouteng Jiangya tablets can inhibit inflammation in the body by inhibiting activation of the TLR4/NF-κB signaling pathway in thoracic aortic endothelial cells, reducing levels of the proinflammatory factors Ang-II and MCP-II, and increasing the level of the anti-inflammatory cytokine Ang(1–7) [36].

In conclusion, our findings reveal that SIRT1 protein expression was significantly enhanced, while that of PTEN was upregulated, in older SHRs treated with PGQYD. These data suggest that PGQYD could delay vascular aging in SHRs through SIRT1–PTEN signaling; however, the mechanisms underlying the mechanism by which PGQYD regulates SIRT1 expression and the specific signaling pathway involved in the regulation of PTEN expression require further clarification.

## Supplementary Information

Supplementary tables

## References

[1] Benschop L, Schelling SJ, Duvekot JJ, Roeters van Lennep JE (2020). Cardiovascular health and vascular age after severe preeclampsia: a cohort study. Atherosclerosis.

[2] Bruno RM, Duranti E, Ippolito C, Segnani C, Bernardini N, Di Candio G (2017). Different impact of essential hypertension on structural and functional age-related vascular changes. Hypertension.

[3] Guarani V, Deflorian G, Franco CA, Krüger M, Phng LK, Bentley K (2011). Acetylation-dependent regulation of endothelial Notch signalling by the SIRT1 deacetylase. Nature.

[4] Bartoli-Leonard F, Wilkinson FL, Schiro A, Inglott FS, Alexander MY, Weston R (2019). Suppression of SIRT1 in diabetic conditions induces osteogenic differentiation of human vascular smooth muscle cells via RUNX2 signalling. Sci Rep.

[5] Yuan Y, Cruzat VF, Newsholme P, Cheng J, Chen Y, Lu Y (2016). Regulation of SIRT1 in aging: roles in mitochondrial function and biogenesis. Mech Ageing Dev.

[6] Shojaee S, Chan LN, Buchner M, Cazzaniga V, Cosgun KN, Geng H (2016). PTEN opposes negative selection and enables oncogenic transformation of pre-B cells. Nat Med.

[7] Jiang Q, Han Y, Gao H, Tian R, Li P, Wang C (2016). Ursolic acid induced anti-proliferation effects in rat primary vascular smooth muscle cells is associated with inhibition of microRNA-21 and subsequent PTEN/PI3K. Eur J Pharmacol.

[8] Zhong GW, Chen MJ, Luo YH (2011). Effect of Chinese herbal medicine for calming Gan (肝) and suppressing hyperactive Yang on arterial elasticity function and circadian rhythm of blood pressure in patients with essential hypertension. Chin J Integr Med.

[9] Zhong GW, Chen MJ, Luo YH, Xiang LL, Xie QY, Li YH (2008). Effects on the vascular remodeling and adiponectin expression in aorta in the spontaneously hypertensive rats by chinese herb mixture method. Chin J Hypertension.

[10] Li Xiangping, Luo Yanhong, Zhong Guangwei, Xiang Linli, Li Yunhui. Pharmacodynamic studies on formula for calming the liver and suppressing yang in treating spontaneous hypertension rats. China J Tradit Chin Med Pharm. 201l; 26: 710–5. Chinese.

[11] Li XP, Luo YH, Zhong GW, Yi T, Xiang LL, Li W (2010). Determination of gastrodin level in serum containing formula for calming the liver and suppressing Yang and investigation of drug serum effects on vascular smooth muscle cell avtivity in rats. Zhongguo Zuzhi Gongcheng Yanjiu Yu Linchuang Kangfu.

[12] Zhong G, Xiang L, Hu J, Yin Y, Chen Q, Fang X (2015). Effect of Pinggan Qianyang recipe on the expression of Tpx II HSP27 and ANXA1 in the hypothalamus of spontaneously hypertensive rats with hyperactivity of liver-YANG syndrome. Zhong Nan Da Xue Xue Bao Yi Xue Ban.

[13] Lvhua YANG, Shazhou ZOU, Yunxia LI (1991). New equipments and methods in measuring systoIic blood pressure and diastolic blood pressure of rats without wounds. Chin J Appl Physiol.

[14] Sun R, Zhang W, Zhong H, Wang L, Tang N, Liu Y (2018). Calcimimetic R568 reduced the blood pressure and improved aortic remodeling in spontaneously hypertensive rats by inhibiting local renin-angiotensin system activity. Exp Ther Med.

[15] Xiong X, Yang X, Liu Y, Zhang Y, Wang P, Wang J (2013). Chinese herbal formulas for treating hypertension in traditional Chinese medicine: perspective of modern science. Hypertens Res.

[16] Li Y, Zhong H, Wu M, Tan B, Zhao L, Yi Q (2019). Decline of p300 contributes to cell senescence and growth inhibition of hUC-MSCs through p53/p21 signaling pathway. Biochem Biophys Res Commun.

[17] Chen K, Sun Z (2018). Activation of DNA demethylases attenuates aging-associated arterial stiffening and hypertension. Aging Cell.

[18] Lee YY, Choi YS, Kim DW, Cheong JY, Song KY, Ryu MS (2020). Mitochondrial nucleoid remodeling and biogenesis are regulated by the p53-p21WAF1-PKCζ pathway in p16INK4a-silenced cells. Aging (Albany NY).

[19] Ding J, Xu K, Sun S, Qian C, Yin S, Xie H (2020). SOCS1 blocks G1-S transition in hepatocellular carcinoma by reducing the stability of the CyclinD1/CDK4 complex in the nucleus. Aging (Albany NY).

[20] Yang G, Zeng X, Li J, Leung CK, Zhang D, Hong S (2019). Protective effect of gastrodin against methamphetamine-induced autophagy in human dopaminergic neuroblastoma SH-SY5Y cells via the AKT/mTOR signaling pathway. Neurosci Lett.

[21] Qing Huo, Qian Zhao, Yu-hua Jiang, Yun-lun. LI (2010). Effects of total alkaloids of Uncaria rhynchophylla on vascular endothelial cell senescence in rats. J Nanjing Univ Traditional Chin Med.

[22] Lei Y, Yang J, Zhao H (2010). Experimental study on extracts from ginseng, notoginseng and chuanxiong for delaying vascular aging in senescent mice. Zhongguo Zhong Xi Yi Jie He Za Zhi.

[23] Wang L, Xu C, Johansen T, Berger SL, Dou Z (2021). SIRT1—a new mammalian substrate of nuclear autophagy. Autophagy.

[24] Lou T, Huang Q, Su H, Zhao D, Li X (2021). Targeting Sirtuin 1 signaling pathway by ginsenosides. J Ethnopharmacol.

[25] Kitada M, Ogura Y, Koya D (2016). The protective role of Sirt1 in vascular tissue: its relationship to vascular aging and atherosclerosis. Aging (Albany NY).

[26] Hekmatimoghaddam S, Dehghani Firoozabadi A, Zare-Khormizi MR, Pourrajab F (2017). Sirt1 and Parp1 as epigenome safeguards and microRNAs as SASP-associated signals, in cellular senescence and aging. Ageing Res Rev.

[27] Guo Y, Xu A, Wang Y (2016). SIRT1 in endothelial cells as a novel target for the prevention of early vascular aging. J Cardiovasc Pharm.

[28] Ortega-Molina A, Efeyan A, Lopez-Guadamillas E, Muñoz-Martin M, Gómez-López G, Cañamero M (2012). Pten positively regulates brown adipose function, energy expenditure, and longevity. Cell Metab.

[29] Garcia-Cao I, Song MS, Hobbs RM, Laurent G, Giorgi C, de Boer VC (2012). Systemic elevation of PTEN induces a tumor-suppressive metabolic state. Cell.

[30] Kälin S, Becker M, Ott VB, Serr I, Hosp F, Mollah MMH (2017). A Stat6/Pten axis links regulatory T Cells with adipose tissue function. Cell Metab.

[31] Ortega-Molina A, Serrano M (2013). PTEN in cancer, metabolism, and aging. Trends Endocrinol Metab.

[32] Ikenoue T, Inoki K, Zhao B, Guan KL (2008). PTEN acetylation modulates its interaction with PDZ domain. Cancer Res.

[33] Wang X, Liu M, Zhu MJ, Shi L, Liu L, Zhao YL (2020). Resveratrol protects the integrity of alveolar epithelial barrier via SIRT1/PTEN/p-Akt pathway in methamphetamine-induced chronic lung injury. Cell Prolif.

[34] Chen G, Tang J, Ni Z, Chen Q, Li Z, Yang W (2015). Antiasthmatic effects of resveratrol in ovalbumin-induced asthma model mice involved in the upregulation of PTEN. Biol Pharm Bull.

[35] Li Yun-Pan, Liu Mei-Yun (2018). Effect of Tianma Gouteng decoction on Ca2+/AMPK/mTOR signaling pathway of vascular endothelial cells in spontaneous hypertensive rats. Zhong Guo Xian Dai Yi Xue Za Zhi.

[36] Xia Li, Yong Zeng, Zhang Wen. Effect of compound Gouteng Jiangya tablet on the inflammatory state of spontaneously hypertensive rats by regulating the TLR4/NF-κB signaling pathway. Hu Nan Zhong Yi Yao Da Xue Xue Bao. 2019;39:168–72.Chinese.

