# Supplementary material for: Effect of the traditional Chinese medicine Pinggan-Qianyang decoction on SIRT1–PTEN signaling in vascular aging in spontaneously hypertensive rats
Source: Hypertens Res. 2021 Jun 29;44(9):1087–98. doi: 10.1038/s41440-021-00682-6 (PMC8418988; doi:10.1038/s41440-021-00682-6)
Supplement: Supplementary file 1 — Supplementary tables [file 41440_2021_682_MOESM1_ESM.doc]

**Supplementary Table 1 Sequences and product size of primers used in this study**

| **Gene:NCBI Reference Sequence** | **Sequences** | **Product size (bp)** |
| --- | --- | --- |
| *SIRT1*:NM_001372090.1 | F:5’-TCAGCTGTTGGCTGACTTCAT-3’  R:5’-TCCCAATGCGATGCTGACTT-3’ | 348 |
| *PTEN*:NM_031606.1 | F:5’-CTCAGCCATTGCCTGTGTGT-3’  R:5’-TCAGGGTGAGCACAAGATACT-3’ | 231 |
| *GAPDH*:NM_017008.4 | F:5’-CAGCCCCAGAGTGTGTATCC-3’  R:5’-GAAGATGCGGTCACCTCACA-3’ | 142 |

F: forward primer. R: reverse primer.

**Supplementary Table 2 Primary antibody information.**

| **Product No.** | **Name** | **MW**  **(kDa)** | **Source/subtype** | **Dilution degree** | **Production company** |
| --- | --- | --- | --- | --- | --- |
| 9475S | SIRT1 | 120 | Rabbit mAb | 1:200 | Cell Signaling Technology, Inc. |
| 9188S | PTEN | 54 | Rabbit mAb | 1:200 | Cell Signaling Technology, Inc. |
| 2527S | p53 | 53 | Rabbit mAb | 1:200 | Cell Signaling Technology, Inc. |
| 2947S | p21 | 21 | Rabbit mAb | 1:200 | Cell Signaling Technology, Inc. |
| 5174S | GAPDH | 37 | Rabbit mAb | 1:300 | Cell Signaling Technology, Inc. |

MW: molecular weight.
